# Efficacy of washing and disinfection in cattle markets in Ireland

**DOI:** 10.1186/s13620-017-0081-1

**Published:** 2017-02-09

**Authors:** Jarlath T. O. Connor, Tracy A. Clegg, Simon J. More

**Affiliations:** 1 Department of Agriculture Food and the Marine, Kildare St, Dublin 2, Ireland; 2 Centre for Veterinary Epidemiology and Risk Analysis, UCD School of Agriculture, Food Science and Veterinary Medicine, University College Dublin, Belfield, Dublin 4, Ireland

**Keywords:** Washing, Disinfection, Cattle, Markets, Ireland

## Abstract

**Background:**

Few studies have reported on the effectiveness of the washing and disinfection methods used in cattle markets in Ireland. Purchasing cattle into recipient herds poses a high biosecurity risk due to the possibility of introducing disease. In Ireland, livestock markets are an important intermediary in the movement of cattle to new herds. Thus disease control strategies need to consider the disease risk associated with moving livestock through markets. Some cattle are also moved directly from markets for slaughter at abattoirs. Washing and disinfection at markets is utilised to reduce faecal contamination in markets, thereby reducing the risk of disease spread among animals and carcass contamination at slaughter. The primary objective of this study was to assess the efficacy of standard washing and disinfection techniques at markets in Ireland in reducing bacterial contamination on internal structures. Total viable counts (TVC) of colony forming units (CFU) were used as indicators of bacterial contamination, which could include pathogens of public and animal health concern. Four hundred and seventy nine samples were taken mainly from pen floors and the TVC enumerated for each sample.

**Results:**

Washing and disinfection was effective at significantly reducing TVCs on floors and metal bars of market holding pens, but residual contamination remained. Washing market pens only (no disinfection), followed by a rest period between batches of cattle (6.5 days) was as effective at reducing TVCs as washing followed by disinfection and a shorter rest period (5.5 days).

**Conclusions:**

Markets are a potential reservoir for microbial contamination with a resultant increased risk of disease spread by cattle moving through markets into new herds, and carcass contamination for cattle moving directly to slaughter. Therefore, market managers need clear advice and guidance on the development of hygiene programmes that are suitable for use in livestock markets.

## Background

In Ireland, approximately 60% of annual cattle movements are through livestock markets. In 2015, 1.7 million cattle moved through 87 registered livestock markets with 92% of those movements to new holdings [1]. Purchasing cattle is a high risk practice for farmers from a biosecurity perspective, due to the possible introduction of disease into the recipient herd [2]. Transportation and commingling of livestock at markets can act as a stressor resulting in immuno-suppression and increased susceptibility to disease [3–5]. Thus, markets have been described as important hubs for the spread of infectious agents [6], and effective control strategies need to consider the risks associated with moving livestock through markets [7].

Apart from the obvious risk of direct spread of infectious agents due to commingling, another risk is faecal contamination of cattle hides from direct contact between animals and/or contact with dirty internal market structures e.g. floors/pens. A potential public health risk may occur when the contaminating faeces contain foodborne pathogens, which subsequently enter the human food chain. In Ireland, some cattle purchased at markets are transported directly for slaughter at abattoirs. Foodborne pathogens that contaminate carcases often originate on the hides of cattle presented for slaughter [8]. While the reported prevalence of important foodborne pathogens in the faeces of livestock varies, even the faeces of healthy livestock can contain those pathogens [9, 10]. Stress associated with transport of livestock to markets may induce the excretion of pathogens such as Salmonellae by livestock [11] with subsequent contamination of internal market structures [3]. Studies on livestock farms and in slaughterhouses lairages have indicated that enteric bacteria may continue to contaminate the environment even after washing and disinfection [12].

In slaughterhouse lairages, where livestock transportation and commingling is similar to that in markets, *Escherichia coli* O157:H7 has been isolated, most frequently on the floors of holding pens [13]. In vitro, foodborne pathogens (*E. coli* 0157, *Salmonella kedougou*, and *Campylobacter jejuni*) have survived for greater than 1 week [14]. If the survival rates for pathogens are commensurate under actual market conditions, then pathogens could be carried over from one batch of animals to another and from one market day to the next. From a foodchain safety perspective, risk mitigation must occur at multiple points to optimise the reduction of risk, with markets an important constituent of the foodchain [15, 16]. The aim of washing and disinfection is to remove organic matter, using physical and water-based washing methods, and to kill remaining micro-organisms using chemical disinfection and natural desiccation [17]. Effective washing and disinfection at markets reduces faecal contamination on internal structures. However, standard washing and disinfection techniques are often complicated by the presence of large quantities of faecal matter, and corroded metal and damaged concrete surfaces.

The primary objective of this study was to assess the efficacy of standard washing and disinfection techniques at markets in reducing bacterial loads on internal market surfaces. A secondary objective included the assessment of the efficacy of three commonly available disinfecting agents, on reducing bacterial load on internal market surfaces.

## Methods

### Markets

The three markets (A, B, C) participating in this study were selected by veterinary officers from the Department of Agriculture, Food and the Marine (DAFM). In all three markets (A, B, C), the holding pen floors and sidebars were constructed of mass concrete and tubular metal, respectively. Washing in all three markets was conducted using high pressure cold water hoses to remove visible dirt. Disinfectant was dispersed directly onto the holding pen floors and sidebars under low pressure using knapsack sprayers.

This paper reports on the outcomes of 2 related studies. Study 1 investigated the efficacy of two markets’ (A and B) washing and disinfection protocols on the bacterial load on holding pen floors and sidebars (Table 1). Study 2 investigated differences in efficacy of three different disinfectants, compared to a control (one pen with no disinfectant), when applied by DAFM officers to nine already washed holding pen floors (one disinfectant per three pens) in Market C (Table 2).

**Table 1:** Washing and disinfection protocols in Markets A & B

Market	Washing Method	Pen rested (days)	Disinfectant	Classification of Disinfectant	Pen rested (days)
A	High pressure, cold water	1	Iosan Farm Disinfectant (Ecolab Ltd, Minnesota, USA)	Iodophor (Phosphoric Acid 15.95%, Iodine 1.75%)	5.5
B	High pressure, cold water	1	Virucidal Extra (Bio Agri Mix, Ontario, Canada)	Oxidising Agent (Potassium peroxymonosulphate 23%)	5.5

**Table 2:** The disinfectants used in Study 2

Market	Washing Method	Pen rested (days)	Disinfectant	Classification of Disinfectant	Pens rested (days)
C (Pens 1–3)	High pressure, cold water	1	Virudine® (DuPont (UK) Ltd, Stevenage, Hertfordshire, UK)	Iodophor (Phosphoric Acid 28%, Iodine 2.8%)	5.5
C (Pens 4–6)	High pressure, cold water	1	Hyperox® (DuPont (UK) Ltd, Stevenage, Hertfordshire, UK)	Oxidising agent (Hydrogen Peroxide, 25%, Peracetic Acid 5%)	5.5
C (Pens 7–9)	High pressure, cold water	1	Virkon® S (Antec International Ltd, Sudbury, Suffolk, UK)	Oxidising Agent (Potassium peroxymonosulphate 21.4%)	5.5
C (Pen 10)	High pressure, cold water	6.5	No disinfectant	–	–

### Sample collection

The markets were visited on market day and ten ‘study pens’ holding cattle were selected at random. Samples from pen floors (Studies 1, 2) and pen bars (Study 1 only) were collected for three different ‘treatments’:

- Treatment 1 (dirty): sampling was conducted immediately after the pens were emptied of cattle;
- Treatment 2 (washed): washing occurred within 3–4 h of the pens being emptied of cattle. Sampling was conducted one hour after washing;
- Treatment 3 (disinfected): Disinfection was conducted the day after the pens (18–24 h) had been emptied of cattle. Post-disinfection sampling occurred the following week, on market day 1–2 h before the pens were reused.

For each of the three treatments, separate samples were collected from 5 randomly selected sites on the pen floor of each study pen. At each selected floor site, vigorous swabbing was conducted over a 20 cm^2^ surface using a sterile pre-moistened sponge swab (3 M Tecra, USA). For Study 1 only, one sample was collected from a single 20 cm^2^ area from the bars of 5 of the study pens. In total 479 samples (449 pen floor samples, 30 pen bar samples for study 1 only) were taken from dirty, washed and disinfected study pens in the 3 markets (A, B, C). Samples were stored at 4 °C during transport to the laboratory, which occurred within 4 h of sample collection.

### Microbiological analysis

Each swab was suspended in 20 ml of Ringer’s solution (quarter strength). Serial ten-fold dilutions were made as per method of Miles and Misra [18], and 100 μl from each dilution was plated in duplicate onto Blood agar and MacConkey agar and incubated at 37 °C ± 1 °C for 24 h. The number of colonies was calculated from the appropriate dilution to give a final total viable count (TVC) of colony forming units (cfu) per swab/20 cm^2^.

### Statistical analyses

The resultant TVC for each sample was transformed into log cfu/cm^2^. Descriptive statistics of the TVC were conducted, by market and treatment. Separate multivariable analyses were carried out for floor and bar samples, with the study pen as the unit of analysis. A repeated measures model was developed using proc mixed in SAS version 9.3 for each study. Correlation between repeated measures of the pen at different treatments was tested by considering different correlations (no correlation, unstructured, compound symmetry and AR1). Market, treatment and an interaction between market and treatment were tested within the model. Differences between the mean TVC at each market during each treatment and between each treatment within each market were tested and differences were adjusted using Bonferroni’s method.

Samples taken from the floors and bars of the same pen (Study 1 only) were compared using a paired Wilcoxon sign rank test and adjusted for multiple comparisons using Bonferroni’s method.

Since there were only a small number pens within each disinfectant trial, and only one control pen, in Study 2 the analysis was descriptive.

## Results

Summary of the log TVC data, by market and treatment, is presented in Table 3.

**Table 3:** Summary of log total viable count (TVC) (log10 cfu/cm^2^) data, by market and treatment

Market	Treatment^a^	N	TVC		
			Mean	Min	Max
A	1	50	7.31	6.00	8.20
	2	50	4.33	3.58	6.52
	3	49	2.71	2.00	4.33
B	1	50	7.84	7.51	8.09
	2	50	4.19	3.49	4.51
	3	50	4.13	3.18	4.61
C	1	50	6.25	5.87	7.15
	2	50	5.69	3.30	6.57
	3	50	3.11	0.00	6.33

^a^Treatment: 1 (dirty), 2 (washed), 3 (disinfected)

### Study 1

#### Floor samples

**Fig. 1:**
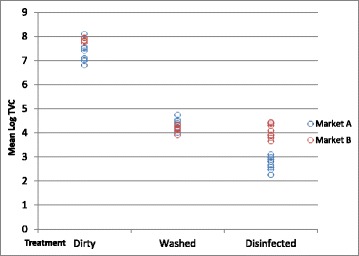
Mean log total viable count (TVC) (log10 cfu/cm^2^) in each study pen in Study 1, by market and treatment

The correlation between each treatment was not significant and a simple model with no correlation was used. The variables: market, treatment and the interaction between market and treatment were all significant (*p* <0.001) in the final model. The difference between the least square means and the adjusted significance of the differences are shown in Table 4. Markets A and B were significantly different at treatment 1 (dirty) and treatment 3 (disinfected) but not at the second treatment (washed) (Fig. 1). In market A, there were significant differences between all treatments, with treatment 3 (disinfected) having the lowest mean TVC. In market B there were significant differences between treatment 1 and the treatments 2 and 3, however treatments 2 and 3 were not significantly different. (Note when median TVC was used instead of mean the results of the significance tests were the same).

**Table 4:** Differences between the least square means of log total viable count (TVC) (log10 cfu/cm^2^) in Study 1 from floors within pens, by market and treatment

Group 1			Group 2			Difference between Group 1 and 2			
Market	Treatment^a^	Least square mean	Market	Treatment^a^	Least square mean	Difference	95% Confidence interval		*P*-value^b^
							Lower	Upper	
A	1	7.31	B	1	7.84	−0.54	−0.88	−0.20	<0.001
A	2	4.33	B	2	4.19	0.14	−0.20	0.48	1.000
A	3	2.70	B	3	4.13	−1.43	−1.77	−1.09	<0.001
A	1	7.31	A	2	4.33	2.98	2.64	3.32	<0.001
A	1	7.31	A	3	2.70	4.61	4.27	4.95	<0.001
A	2	4.33	A	3	2.70	1.63	1.29	1.97	<0.001
B	1	7.84	B	2	4.19	3.66	3.32	4.00	<0.001
B	1	7.84	B	3	4.13	3.72	3.38	4.06	<0.001
B	2	4.19	B	3	4.13	0.06	−0.28	0.40	1.000

^a^Treatment: 1 (dirty), 2 (washed), 3 (disinfected)

^b^Adjusted to account for multiple comparisons using Bonferroni’s adjustment

### Bar samples

The final model for bar samples was similar to that for floor samples (Table 5). There was a borderline significant difference between markets A and B at treatment 2 (washed) and no significant difference at treatment 3 (disinfected). In both markets there were significant differences between treatments 1 and the other two treatments, however there were no differences between treatments 2 and 3 in either market.

**Table 5:** Differences between the least square means of log total viable count (TVC) (log10 cfu/cm^2^) in Study 1 from bars within pens, by market and treatment

Group 1			Group 2			Difference between Group 1 and 2			
Market	Treatment^a^	Least square mean	Market	Treatment^a^	Least square mean	Difference	95% Confidence interval		*P*-value^b^
							Lower	Upper	
A	1	5.85	B	1	7.87	−2.02	−2.75	−1.29	<0.001
A	2	3.10	B	2	4.11	−1.01	−1.70	−0.32	0.052
A	3	2.95	B	3	3.78	−0.83	−1.52	−0.14	0.186
A	1	5.85	A	2	3.10	2.75	2.02	3.48	<0.001
A	1	5.85	A	3	2.95	2.90	2.17	3.63	<0.001
A	2	3.10	A	3	2.95	0.15	−0.54	0.84	1.000
B	1	7.87	B	2	4.11	3.76	3.07	4.45	<0.001
B	1	7.87	B	3	3.78	4.09	3.40	4.78	<0.001
B	2	4.11	B	3	3.78	0.33	−0.36	1.02	1.000

^a^Treatment: 1 (dirty), 2 (washed), 3 (disinfected)

^b^Adjusted to account for multiple comparisons using Bonferroni’s adjustment

### Difference between bar and floor samples

There was no significant difference (Wilcoxon sign rank test, Bonferroni adjusted *p*-value > = 0.375) between the samples taken from the pen floors and bars at each of the 3 stages in either market.

### Study 2

**Fig. 2:**
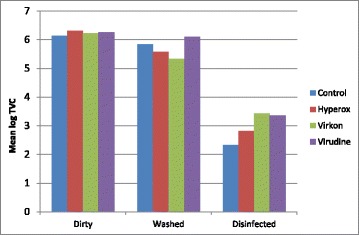
Mean log total viable count (TVC) (log10 cfu/cm^2^), for each pen in Study 2, by treatment and disinfectant (‘Disinfected’ control is resting period and desiccation for 6.5 days)

The mean log TVCs of the pens at each treatment are shown in Fig. 2. After disinfection the control pen had the lowest log TVC compared to the 3 disinfected groups of pens.

## Discussion

There are few published studies on the efficacy of washing and disinfection practices in markets. While washing coupled with mechanical cleaning is reportedly the most efficient way to remove micro-organisms [19], in our study mechanical washing was not employed in conjunction with high pressure cold water hosing as part of the standard market cleansing protocol in any of our three markets. It is possible that power hosing of pen floors may cause splashing of faecal material, resulting in the dispersion of contaminants. Lowering the water spray pressure during hosing may limit the spread of contamination due to the reduced generation of suspended aerosols [20]. The addition of detergents to wash water can greatly reduce contamination by aerosols [21]. In livestock housing the inclusion of detergent in a cleaning regime can significantly reduce bacterial loads on concrete and metal [17]. However, many Irish markets do not have the facilities to deliver detergents at the washing stage. The application of steam following high pressure washing can be more effective at reducing *Enterobacteriaceae* on pen floors than either pressure washing alone, or in combination with detergents [22]. Similarly, directed mist application with peroxygen disinfectant may be an effective means of environmental disinfection compared to traditional washing and disinfection in certain circumstances [23]. The feasibility and applicability of applying steam or misted disinfectant solutions on pressure washed floors has not yet been established for markets in Ireland.

The results of study 1 indicate that high-pressure cold water hosing is effective in significantly reducing, but not eliminating, TVCs in markets. However, the use of disinfectants following hosing had mixed results on significantly reducing TVCs. Such results may be explained by the use of inappropriate or incorrectly constituted disinfectants, and/or inadequate operator care in the dispersion of the disinfectant. The effectiveness of disinfection is reduced unless all surfaces have previously been thoroughly washed to remove interfering materials [24]. Washing is therefore extremely important as part of a two-stage cleaning and disinfection programme. In Ireland, markets are often ageing structures, as few new markets have been built in recent years, resulting in floor and internal structure surfaces that are sometimes rough and worn. As smoother surfaces tend to be less contaminated than rougher ones and easier to wash [25], older markets with worn surfaces are often difficult to effectively wash and disinfect. While the pens appeared visually clean after washing, this does not imply the effective reduction of bacteria and especially *Enterobacteriaceae* [26].

Furthermore, study 1 showed that there was no significant difference in TVC between pen floors and pen bars before or during the course of the cleansing process. This finding suggests that pen floors and pen bars should receive the same attention during cleansing, even if pen floors often visibly appear more faecally contaminated. Washing and disinfecting pen bars is more time consuming than solely focusing on the pen floor, resulting in increased staff time and costs.

Due to the small sample size in study 2, little can be concluded from the use of three different commonly available disinfectants versus a control pen. The TVCs recovered post disinfection were similar for the three disinfectants thereby eliminating the need to discuss the efficacy of the individual disinfectant families utilized. In the control pen which was not disinfected but rested for six days, the TVCs were not significantly different from those of the disinfected pens. This is in agreement with other studies that attribute desiccation as the main cause of microbial death during the rest period [17]. Further study could be useful in addressing the efficacy of disinfectants compared to the use of an optimal rest period following washing. Additionally, there appears to be potential residual bacterial contamination which persists after washing and disinfection of a similar range to that recovered from the hides of cattle at slaughter [27]. Residual bacterial contamination in markets may therefore be a source of carcass contamination at slaughter especially for those cattle moved quickly to slaughter from markets.

Little literature exists on the effect of washing and disinfection on reducing the recovery of significant foodborne pathogens, such as *E. coli and Salmonellae*, from market infrastructures. Our study indicates that residual environmental contamination with bacteria is likely in cattle markets, even after washing and disinfection. Such contamination is likely to persist until subsequent sales days. While we used TVCs as an indicator for bacterial contamination in markets, further study is required to identify the proportion of residual contamination containing potential foodborne pathogens.

An important factor in influencing the contamination of beef carcasses during the slaughter process is the pathogen load on live animals entering the abattoir, which in turn depends on environmental exposure to contaminants [27]. Lack of en route hygiene measures, especially at markets, can have a profound effect on microbial levels at slaughter and may pose considerable risks to product quality [28].

## Conclusions

This study highlights that markets are a potential reservoir for microbial contamination, thereby increasing the risk of disease spread among cattle transiting markets, and increasing the risk of carcass contamination for cattle moving directly to slaughter from markets. The total elimination of faecal contamination at markets is impractical, but washing and disinfection is a practical control method to reduce pathogen levels. Therefore, market managers need clear advice and guidance on the development of hygiene programmes that are suitable for use in livestock markets. Further research is needed to establish practical and effective hygiene programmes that are suitable for use in livestock markets in Ireland.
